# CARDIOVASCULAR RISK MODERATES THE EFFECT OF RESISTANCE TRAINING ON PHYSICAL PERFORMANCE IN OLDER ADULT WOMEN

**DOI:** 10.1093/geroni/igz038.2396

**Published:** 2019-11-08

**Authors:** Lauren Marcotte, Cindy Barha, Teresa Liu-Ambrose

**Affiliations:** University of British Columbia, Vancouver, British Columbia, Canada

## Abstract

We aimed to examine whether the Framingham Cardiovascular Risk Profile Score (FCRP) moderates the effect of progressive resistance training (RT) on mobility in older adult women. This is an exploratory analysis of a single-blind, 12-month randomized controlled trial in 155 omen, aged 65 to 75 years old, who were randomized to: 1x/week progressive RT; or 2x/week progressive RT program; or 2x/week balance and tone (BAT). At baseline and trial completion, mobility was measured using the Short Physical Performance Battery (SPPB). The SPPB is a composite measure of usual gait speed, standing balance, and sit to stand performance; scores < 9/12 are indicative of functional decline. Baseline 10-year cardiovascular risk was calculated using the FCRP. Participants were classified as either low risk (<16.5% FCRP score; LCVR) or high risk ≥16.5% FCRP score; HCVR). A complete case analysis (n=126) was conducted using a two-way analysis of covariance (ANCOVA) to evaluate the interaction effect of group by FCRP risk on SPPB scores at trial completion; baseline SPPB scores and age in years were entered as covariates. There was a significant interaction effect (F(1,126)=3.74, p=0.027). At trial completion, both 1x/RT and 2x/RT participants with HCVR demonstrated greater SPPB scores than those with LCVR (11.59 vs. 11.38 for 1x/week; 11.86 vs 11.46 for 2x/week). In contrast, BAT participants with HCVR demonstrated worse SPPB scores than those with LCVR (11.18 vs 11.66). Our data suggest that RT may be more efficacious for improving mobility in older women with higher cardiovascular risk than women with lower risk.

